# Improved automatic identification of isolated rapid eye movement sleep behavior disorder with a 3D time‐of‐flight camera

**DOI:** 10.1111/ene.15822

**Published:** 2023-05-23

**Authors:** Matteo Cesari, Laurenz Ruzicka, Birgit Högl, Abubaker Ibrahim, Evi Holzknecht, Anna Heidbreder, Melanie Bergmann, Elisabeth Brandauer, Heinrich Garn, Bernhard Kohn, Ambra Stefani

**Affiliations:** ^1^ Department of Neurology Medical University of Innsbruck Innsbruck Austria; ^2^ Competence Unit Sensing and Vision Solutions AIT Austrian Institute of Technology GmbH Vienna Austria

**Keywords:** alpha‐synucleinopathy, computerized method, nearable sensor, prodromal, upper extremities

## Abstract

**Background and purpose:**

Automatic 3D video analysis of the lower body during rapid eye movement (REM) sleep has been recently proposed as a novel tool for identifying people with isolated REM sleep behavior disorder (iRBD), but, so far, it has not been validated on unseen subjects. This study aims at validating this technology in a large cohort and at improving its performances by also including an analysis of movements in the head, hands and upper body.

**Methods:**

Fifty‐three people with iRBD and 128 people without RBD (of whom 89 had sleep disorders considered RBD differential diagnoses) were included in the study. An automatic algorithm identified movements from 3D videos during REM sleep in four regions of interest (ROIs): head, hands, upper body and lower body. The movements were divided into categories according to duration: short (0.1–2 s), medium (2–15 s) and long (15–300 s). For each ROI and duration range, features were obtained from the identified movements. Logistic regression models using as predictors the features from one single ROI or a combination of ROIs were trained and tested in a 10‐runs 10‐fold cross‐validation scheme on the task of differentiating people with iRBD from people without RBD.

**Results:**

The best differentiation was achieved using short movements in all four ROIs (test accuracy 0.866 ± 0.007, test F1 score = 0.783 ± 0.010). Single group analyses showed that people with iRBD were distinguished successfully from subjects with RBD differential diagnoses.

**Conclusions:**

Automatic 3D video analysis might be implemented in clinical routine as a supportive screening tool for identifying people with RBD.

## INTRODUCTION

Rapid eye movement (REM) sleep behavior disorder (RBD) is a parasomnia characterized by abnormal muscle tone (REM sleep without atonia, RWA) and dream‐enacting behaviors during REM sleep [1]. Isolated RBD (iRBD) is recognized as an early stage alpha‐synucleinopathy [2, 3]. Its correct diagnosis is fundamental in the light of upcoming disease‐modifying neuroprotective treatments [4].

According to the third edition of the International Classification of Sleep Disorders (ICSD‐3) [1], polysomnographic demonstration of RWA is required for RBD diagnosis, whereas video‐polysomnography (v‐PSG) is required to make a definite RBD diagnosis according to the guidelines of the International RBD Study Group, as at least one RBD episode needs to be demonstrated in the video [5]. Both manual RWA quantification and visual video inspection are time‐consuming processes. Therefore, these procedures would highly benefit from the availability of automatic methods. In particular, automatic video analysis would be highly beneficial, as abnormal movement activity is an intuitively understandable biomarker of disease. Whilst for RWA quantification several automatic methods have been proposed [6, 7, 8, 9, 10, 11], only two previous studies from our group have investigated automatic video analysis to identify RBD [12, 13]. In both studies, videos were recorded with a 3D time‐of‐flight (TOF) sensor, allowing movements to be identified and quantified with high precision. The first study showed that movements in the lower limbs could differentiate 40 people with iRBD from 64 people with other disorders with motor activity during sleep with 90.4% accuracy [12]. In the second pilot study, the combination of movements in the upper body (i.e., above the hips) and lower limbs differentiated 20 people with iRBD from 24 people with sleep‐related breathing disorders with increased accuracy compared to the analysis of movements in the lower limbs only [13]. Both these studies analyzed only movements included in manually identified REM sleep and the body regions were manually identified.

Although important for paving the way, these two studies have limitations. First, in both studies no validation performances on unseen subjects were calculated; thus they did not provide an unbiased estimation of identification performances. Secondly, the sample size of the second study was small; therefore confirmation on a larger cohort is needed. Thirdly, the upper body region used in the second study included head, trunk and upper extremities without further distinction. Based on previous literature showing that muscular activity in the upper extremities allows sensitive and specific identification of people with RBD [14, 15, 16, 17] and that neck myoclonus (also known as head jerk) is more prevalent in people with RBD than in those without RBD [18], it can be hypothesized that identification of people with RBD could be improved by differentiating the involved body parts.

This study aims at overcoming these limitations by validating 3D video analysis of movements occurring in manually scored REM sleep and in semi‐automatically identified body regions (head, upper extremities, upper body and lower body) in a large cohort as a novel technology for objective identification of people with iRBD.

## METHODS

### Subjects and v‐PSG recordings

In this prospective study, a total of 181 people were included who underwent v‐PSG for one night at the Sleep Disorders Unit, Department of Neurology, Medical University of Innsbruck. v‐PSGs were recorded according to the American Academy of Sleep Medicine recommendations [19]. Sleep experts manually scored sleep stages and respiratory events according to international criteria [19]. Periodic leg movements (PLMs) were automatically scored with a validated software [20] and RWA was quantified for all subjects with the SINBAR score [16] with semi‐automatic software [8].

Sleep diagnoses were made according to the ICSD‐3 [1] and RBD was diagnosed according to recent international guidelines [5]. The cohort included 53 people with iRBD, 51 with sleep‐related breathing disorder (SRBD) (i.e., people with apnea−hypopnea index ≥15/h or people with apnea−hypopnea index ≥5/h and with symptoms as described by the ICSD‐3 [1]), 20 with restless legs syndrome (RLS), 20 with insomnia, 12 presenting PLMs during sleep without any associated sleep disorder (i.e., PLM index ≥15/h), six with non rapid eye movement (NREM) parasomnia and 19 for whom no relevant sleep disorder was identified. Table 1 shows the sleep and demographic information of the subjects included in the study. The people not diagnosed with iRBD are merged in the category “no‐RBD”. Tables S1–S6 present detailed demographic and sleep information of the different groups.

**TABLE 1:** Demographic and sleep information

Parameter	iRBD*	no‐RBD	*p* value
*N*	53	128	−
Males (%)	84.9	61.7	**0.002**
Age (years)	65.5 ± 9.5	54.3 ± 13.4	**<0.001**
REM duration (min)	66.1 ± 31.4	71.3 ± 31.8	0.320
AHI (/h)	6.8 [2.7–13.2]	6.2 [2.0–20.5]	0.963
AHI in REM (/h)	7.6 [1.7–14.0]	6.7 [1.1–26.7]	0.839
PLMS index (/h)	28.1 [16.5–58.4]	13.3 [3.2–32.6]	**<0.001**
PLMS index in REM (/h)	38.0 [19.0–73.0]	2.0 [0.0–9.0]	**<0.001**
PAP therapy during v‐PSG (%)	34.0	10.2	**<0.001**
Taking antidepressants (%)	47.2	17.2	**<0.001**
SINBAR (%)	61.4 [48.4–74.4]	15.2 [10.7–23.9]	**<0.001**

*Note*: Values are shown as mean ± standard deviation if normally distributed and as median [interquartile range] otherwise. For normally distributed variables, *t* tests were used to compare groups; otherwise Mann−Whitney *U* tests were employed. Categorical variables were compared with chi‐squared tests. Significant *p* values (<0.05) are highlighted in bold.

Abbreviations: AHI, apnea−hypopnea index; iRBD, isolated rapid eye movement sleep behavior disorder; PAP, positive air pressure; PLMS, periodic limb movement during sleep; RBD, rapid eye movement sleep behavior disorder; REM, rapid eye movement; SINBAR, Sleep Innsbruck Barcelona; v‐PSG, video‐polysomnography.

* List of comorbidities of people with iRBD: (i) seven also had diagnosis of sleep‐related breathing disorder (SRBD); (ii) 17 had periodic limb movements (PLMs) during sleep; (iii) 13 had a diagnosis of SRBD and had PLMs during sleep; (iv) four had diagnosis of restless legs syndrome (RLS) and presented PLMs during sleep; (v) four had diagnosis of RLS, SRBD and had PLMs during sleep; (vi) one had RLS and SRBD diagnoses; (vii) one had insomnia and presented PLMs during sleep; (viii) two had insomnia, RLS and presented PLMs during sleep; (ix) one had insomnia, RLS, SRBD and PLMs during sleep; (x) one had diagnoses of insomnia and SRBD; and (xi) two did not have any additional sleep disorder.

### 3D video recording and movement detection

Simultaneously with the v‐PSG, an infrared 3D video was recorded with a Microsoft Kinect v2 (Microsoft Corporation), which uses a TOF sensor. This device was mounted on the ceiling, centered above the bed. At a resolution of 30 frames per second, the TOF sensor obtained depth images (resolution 512 × 424 pixels), which contained information on the distance between the sensor and the surface reflecting the infrared light (i.e., the bed or the patient) emitted from the sensor itself.

**FIGURE 1:**
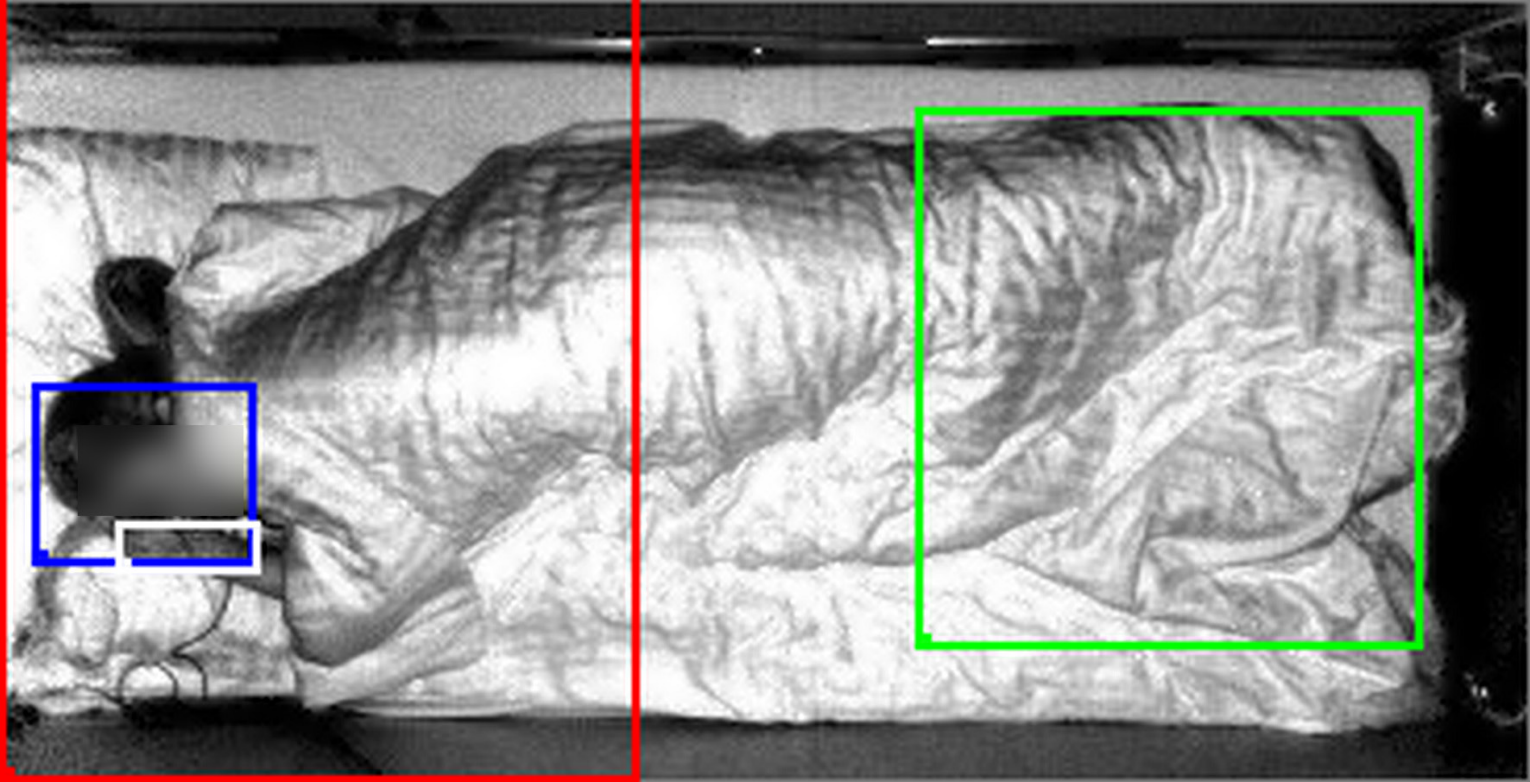
Regions of interest (ROIs) investigated in this study. The figure shows an example of the different ROIs: lower body (green), hands (white), head (blue), upper body (red).

To identify movements from depth images, a pipeline previously described was applied [12, 21]. Compared with our previous work where only lower limb movements were analyzed [12], further regions of interest (ROIs) (Figure 1) were added. More specifically, movements in the lower body (i.e., the region including the knees and the area below them, as previously proposed [12]), head, hands (including one or both hands) and upper body (region above the hip) were identified. Whilst upper and lower body ROIs were manually identified, head and hands ROIs were dynamically identified with a validated deep neural network as described elsewhere [22]. Such neural network was trained to identify head and hands when not covered by the blankets. When head and hands were automatically identified, their movements were not considered as upper body movements. On the other hand, if the network did not recognize head and/or hands, movements associated with them were still counted as movements in the upper body ROI. As an identification of the whole upper extremities and differentiation of their movements was not possible, movements of the hands were used as a way to quantify movements in the upper extremities.

### Feature extraction

The identified movements were divided into categories according to different duration intervals: (i) between 0.1 and 2 s (short movements), (ii) between 2 and 15 s (movements of medium duration) and (iii) between 15 and 300 s (movements of long duration). Two movements were considered as separate in the case of an inter‐movement interval above 1 s. For each ROI and duration interval, two features were calculated: the 3D rate (defined as the number of identified movements in REM sleep per hour of REM sleep) and the 3D ratio (defined as the total movement duration time in REM sleep per hour of REM sleep, multiplied by a scaling factor of 3600). To compute these features, the manual annotations of REM sleep from the scored v‐PSG were used.

### Classification

In a 10‐fold cross‐validation scheme (repeated for 10 runs with different random seeds), logistic regression classifiers were trained and tested with ridge regularization to distinguish people with iRBD from the no‐RBD group (classification iRBD vs. no‐RBD). For each duration interval, seven different classifiers were trained and tested, which used as predictor features the 3D rate and 3D ratio from (i) head ROI only, (ii) hands ROI only, (iii) upper body ROI only, (iv) lower body ROI only, (v) head, hands and upper body ROIs, (vi) head, hands and lower body ROIs, and (vii) head, hands, upper body and lower body ROIs. In other words, for the classifications (i)‐(iv), two features were used as predictors (i.e., the 3D rate and 3D ratio from one single ROI), for classifications (v) and (vi), six features were used as predictors (i.e., the 3D rate and 3D ratio from three ROIs), and finally for classification (vii) a total of eight features were used as predictors (i.e., the 3D rate and 3D ratio from all ROIs).

During cross‐validation, each feature was normalized to the 5th and 95th percentile of the feature values in the training set. Accuracy, F1 score, sensitivity, specificity, positive and negative predictive values for identification of people with iRBD were calculated. To understand whether there were differences in the classification between iRBD and other specific groups, the test performances were calculated separately for distinguishing people with iRBD from (i) people with SRBD (classification iRBD vs. SRBD), (ii) people with RLS or PLMs without any other associated disease (classification iRBD vs. RLS/PLMS) and (iii) people with insomnia or people without any relevant sleep disorder (classification iRBD vs. INS/NRSD).

To understand whether there were differences in the classification iRBD versus no‐RBD between movements with different duration ranges, the obtained test accuracy and F1 score were compared with signed Wilcoxon rank tests corrected with Benjamini−Hochberg correction (*q* = 0.05). Furthermore, with the same statistical approach the values of test accuracy and F1 score of the classifications iRBD versus SRBD, iRBD versus RLS/PLMS and iRBD versus INS/NRSD were compared, to understand whether there were differences in the classification performances between iRBD and different groups.

## RESULTS

Table 2 shows the values of the features for people with iRBD and the no‐RBD group for different duration intervals and ROIs. All features were significantly different between people with iRBD and the no‐RBD group in all ROIs only for the interval 0.1–2 s.

**TABLE 2:** Features values

ROI	Feature	0.1–2 s			2–15 s			15–300 s		
		iRBD	no‐RBD	*p* value	iRBD	no‐RBD	*p* value	iRBD	no‐RBD	*p* value
HE	3D rate	15.85 [9.17, 30.17]	7.46 [2.91, 11.71]	**<0.001**	5.43 [3.18, 8.01]	4.05 [1.43, 6.48]	**0.022**	0 [0, 0]	0 [0, 0]	0.059
	3D ratio	12.02 [8, 19.91]	6.17 [2.51, 9.9]	**<0.001**	25.8 [13.27, 34.59]	21.1 [5.31, 33.76]	0.091	0 [0, 0]	0 [0, 0]	0.065
HAs	3D rate	17.38 [6.63, 33.32]	7.19 [2.37, 15.33]	**<0.001**	3.96 [1.37, 11.97]	1.55 [0, 5.86]	**0.001**	0 [0, 0]	0 [0, 0]	‐
	3D ratio	13.52 [4.77, 27.41]	5.01 [1.52, 11.82]	**<0.001**	14.43 [4.64, 41.96]	5.03 [0, 21.81]	**0.001**	0 [0, 0]	0 [0, 0]	‐
UB	3D rate	31.88 [18.65, 82.1]	9.64 [4.44, 14.96]	**<0.001**	9.8 [5.6, 16.49]	5.11 [2.37, 9.85]	**<0.001**	0.47 [0, 1.69]	0 [0, 1.14]	0.074
	3D ratio	26.67 [12.34, 53.09]	7.69 [3.26, 11.63]	**<0.001**	50.19 [28.69, 75.82]	30.28 [12.31, 56.79]	**0.002**	9.72 [0, 32.37]	0 [0, 19.36]	0.074
LB	3D rate	44.12 [32.93, 63.73]	10.90 [5.94, 19.17]	**<0.001**	51.75 [34.57, 82.24]	16.01 [10.10, 25.01]	**<0.001**	2.23 [0.38, 4.34]	0.94 [0, 2.23]	**0.003**
	3D ratio	50.03 [38.43, 77.06]	12.45 [6.54, 21.24]	**<0.001**	247.5 [154.8, 379.59]	81.71 [45.67, 124.39]	**<0.001**	45.34 [7.38, 98.07]	17.88 [0, 49]	**0.002**

*Note*: For each region of interest (ROI) and interval duration the values of the features 3D rate and 3D ratio are shown as median and interquartile range for iRBD patients and the no‐RBD group. Mann−Whitney *U* tests are used to compare the values and significant *p* values (<0.05) are highlighted in bold.

Abbreviations: HAs, hands; HE, head; iRBD, isolated rapid eye movement sleep behavior disorder; LB, lower body; RBD, rapid eye movement sleep behavior disorder; ROI, region of interest; UB, upper body.

**FIGURE 2:**
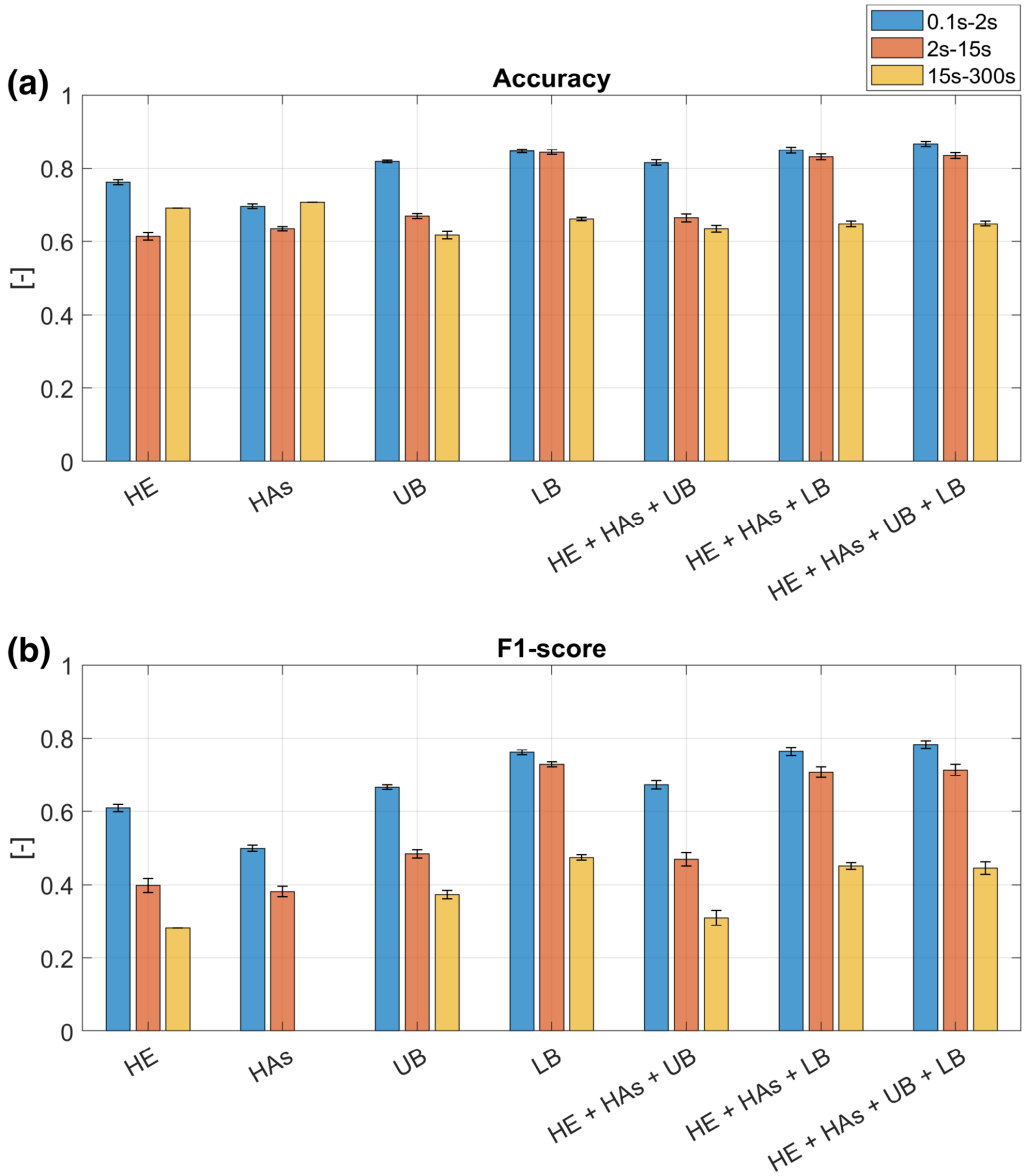
Test accuracy (a) and F1 score (b) for the classification iRBD versus no‐RBD group. The mean and standard deviation (across the 10 runs of 10‐fold cross‐validation) values of accuracy and F1 score are reported for each interval duration and when considering as predictor features the 3D rate and 3D ratio from (i) head region of interest (ROI) only (HE), (ii) hands ROI only (HAs), (iii) upper body ROI only (UB), (iv) lower body ROI only (LB), (v) head, hands and upper body ROIs (HE+HAs+UB), (vi) head, hands and lower body ROIs (HE+HAs+LB) and (vii) head, hands, upper body and lower body ROIs (HE+HAs+UB+LB).

Table S7 shows the training performances for distinguishing people with iRBD from the no‐RBD group. Table S8 shows the detailed test performances and Table S9 the statistical comparison of accuracy and F1 score values. To facilitate comparison between different duration intervals, Figure 2 shows test accuracy and F1 score values for all classifiers. People with iRBD were distinguished from the no‐RBD group with significantly higher accuracy and F1 score when using features extracted from movements with short duration (0.1–2 s) than when using features from movements with medium or long duration. This pattern was observed across all classifiers using either features from single ROIs only or multiple features from different ROIs, except for the lower body ROI for which the accuracy did not differ significantly between short and medium duration movements. Thus, short movements were found to best differentiate people with iRBD from the no‐RBD group. Because of this finding, the focus was on analyzing results obtained considering only short movements.

**FIGURE 3:**
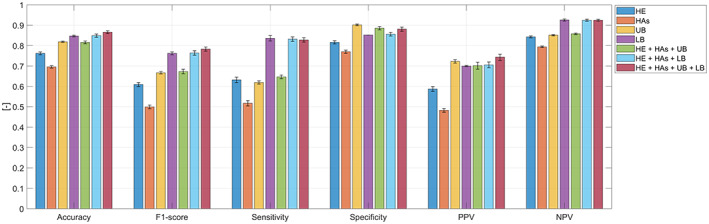
Test classification performances (iRBD vs. no‐RBD) considering short movements (0.1–2 s). For each measure of performance, the values reported as mean and standard deviation across the 10 runs of 10‐fold cross‐validation are obtained by using as predictor features the 3D rate and 3D ratio from (i) head region of interest (ROI) only (HE), (ii) hands ROI only (HAs), (iii) upper body ROI only (UB), (iv) lower body ROI only (LB), (v) head, hands and upper body ROIs (HE+HAs+UB), (vi) head, hands and lower body ROIs (HE+HAs+LB) and (vii) head, hands, upper body and lower body ROIs (HE+HAs+UB+LB). NPV, negative predictive value; PPV, positive predictive value.

Figure 3 shows all test performance measures for the discrimination of iRBD people from the no‐RBD group obtained by the seven classifiers trained and tested when considering only short movements. Table S10 shows the corrected *p* values obtained from statistically comparing the accuracy and F1 scores presented in Figure 3. When considering features from all four ROIs (i.e., head, hands, upper body and lower body), iRBD was distinguished from no‐RBD with an accuracy of 0.866 ± 0.007 and F1 score of 0.783 ± 0.010, which were significantly higher than the accuracy and F1 score values obtained by considering features from single ROIs or the combination of three ROIs. No significant differences were observed between the accuracy and F1 score values obtained by using features from the lower body alone and the ones obtained by using features from the head, hands and lower body. The same was observed for the upper body. The highest average sensitivity (0.836) and negative predictive value (0.926) were obtained when considering features only from movements in the lower body ROI. The highest average specificity (0.902) was obtained when considering movements in the upper body. The highest average positive predictive value (0.743) was reached when considering movements from the four ROIs as well as only movements from the upper body.

**FIGURE 4:**
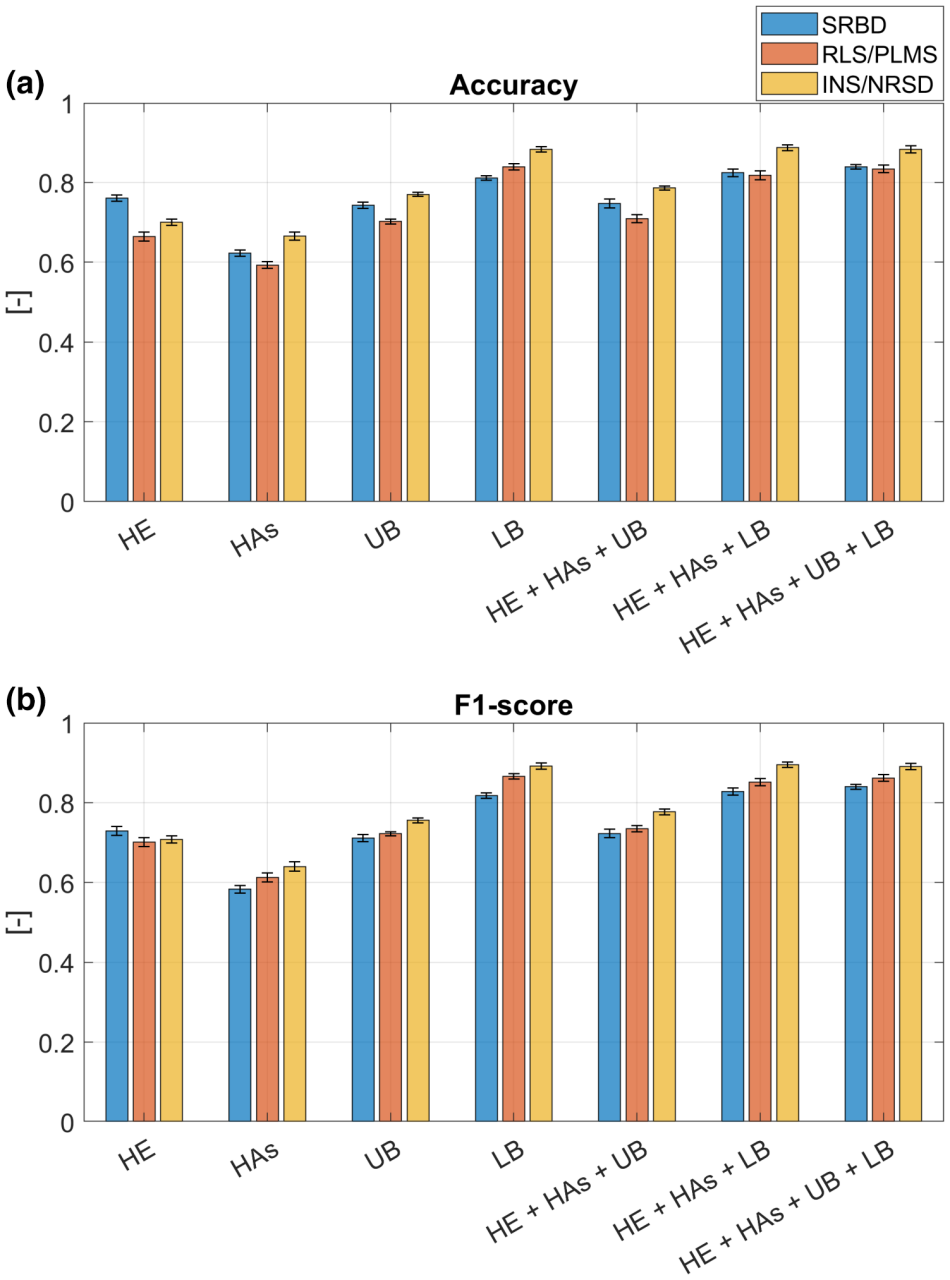
(a) Test accuracy and (b) F1 score when classifying iRBD versus distinct groups using short movements. The mean and standard deviation values of accuracy and F1 score across the 10 runs of 10‐fold cross‐validation are reported for each classification (i.e., iRBD vs. SRBD, iRBD vs. RLS/PLMS and iRBD vs. INS/NRSD) and when considering as predictor features the 3D rate and 3D ratio from (i) head region of interest (ROI) only (HE), (ii) hands ROI only (HAs), (iii) upper body ROI only (UB), (iv) lower body ROI only (LB), (v) head, hands and upper body ROIs (HE+HAs+UB), (vi) head, hands and lower body ROIs (HE+HAs+LB) and (vii) head, hands, upper body and lower body ROIs (HE+HAs+UB+LB). INS, insomnia; iRBD, isolated rapid eye movement sleep behavior disorder; NRSD, non‐relevant sleep disorder; PLMS, periodic limb movements during sleep; RLS, restless legs syndrome; SRBD, sleep‐related breathing disorder.

The test performances of the classifications of people with iRBD versus people with SRBD, RLS/PLMS and INS/NRSD when considering short movements are shown in detail in Table S11. Figure 4 shows the accuracy and F1 score values, and the corrected *p* values of the statistical analyses are reported in Table S12. Subjects with INS/NRSD could be significantly better differentiated from people with iRBD compared to people with SRDB or RLS/PLMS when using features from multiple ROIs, hands ROI only, upper body ROI only and lower body ROI only. For the classifier using the features from head ROI only, significantly higher F1 score and accuracy were obtained for the classification iRBD versus SRBD compared to the other two classifications. For the classification iRBD versus SRBD, the highest average accuracy (0.839) and F1 score (0.840) were achieved when considering movements from the four ROIs. For the classification iRBD versus RLS/PLMS, the highest average accuracy (0.839) and F1 score (0.866) were achieved when considering movements from the lower body ROI only. Finally, for the classification iRBD versus INS/NRSD, the highest average accuracy and F1 score (0.888 and 0.895, respectively) were achieved when movements in the head, hands and lower body ROIs were considered.

Additional analyses for the classification considering short movements from all four ROIs showed that the classification was not influenced by differences in age and gender between groups (Table S13) and that there was significant correlation (Spearman correlation coefficient 0.645, *p* value < 0.001) between the test probability of iRBD from the logistic regression models and the SINBAR RWA index (Figure S1).

## DISCUSSION

In this study, the aim was to improve and advance the previously proposed automatic analysis of 3D videos recorded with a TOF sensor for identification of people with iRBD [12, 13]. Compared to the previous approach, movements were separately identified in multiple body parts, the methodology was validated on unseen subjects in a large cohort and the performances were assessed with a 10‐fold cross‐validation approach. It was shown that (i) short movements with duration 0.1–2 s best differentiated people with iRBD from the no‐RBD group; (ii) combining movements identified in the lower body with movements in the upper body, head and hands led to significantly improved accuracy and F1 score for identification of people with iRBD; and (iii) subjects with iRBD could be distinguished with the best performances from people with insomnia or without any relevant sleep disorders, but high accuracy and F1 score could be achieved also for differentiating them from subjects with sleep‐related breathing disorder, RLS or PLMs.

The study confirms that short movements (i.e., jerks) are the true hallmark of RBD, as previously shown in visual video analyses [23, 24] and as was previously shown when analyzing lower limb movements only [12].

When considering the differentiation of people with iRBD from the no‐RBD group, a significant increase in accuracy and F1 score was shown when combining movements from the lower body with those in the head, hands and upper body. In particular, specificity and positive predictive value for iRBD identification increased when also including movements identified in the head, hands and upper body. This is in line with our previous preliminary findings [13], as well as with previous literature showing improved identification of people with RBD when recording muscle activity in the upper extremities [15, 16, 17] and with the increased frequency of neck myoclonus (i.e., head jerks) in subjects with RBD [18]. However, when considering only movements in the head, hands and upper body (thus excluding the lower body), people with iRBD were differentiated from the no‐RBD group with significantly lower accuracy and F1 score than when movements in the lower body only were considered. In particular, the classifier using only movements in the head, hands and upper body showed low sensitivity to discriminate people with iRBD. The cause of this low sensitivity could be multifold. First, there is probably an optimization issue in the identification of movements from 3D video in the head, hands and upper body. In fact, whilst the threshold used to identify movements in the lower body has been optimized in previous studies for identifying leg movements [25], the thresholds used to identify movements in the upper body, head and hands were not optimized against manually annotated movements but only with a trial and error procedure on eight recordings and in random portions of REM sleep. Furthermore, despite the application of a respiration filter to remove respiratory movements [26], it is possible that movements in the upper body ROI might still include respiration‐related movements, thus confounding the classification. Finally, there was a significant difference in the use of positive airway pressure (PAP) between people with iRBD and the no‐RBD group and it is not clear to what extent the movements of the PAP tube could have influenced the automatic analysis of upper body movements.

It is also worth noticing that, in the classification iRBD versus no‐RBD, the lowest accuracy and F1 score were achieved when considering hands only. This could be because the deep neural network had moderate hands detection accuracy (on average 41.6%). This may be caused by the variable and often changing position of the hands during the night, as well as the technical difficulties of identifying a small area of a few pixels. These low performances should not be seen as contradicting the literature showing the importance of the upper extremities to correctly diagnose RBD [14, 15, 16, 17] but indicate instead the need for future improvements to better identify hands from 3D videos.

In the no‐RBD group, patients with differential diagnoses to RBD were included. In particular, (i) people with SRBD, as movements at the end of an apnea might mimic RBD behaviors [27]; (ii) subjects with RLS or PLMs during sleep as leg movements might also mimic RBD [28]; and (iii) people with NREM parasomnia, because of the abnormal behaviors characterizing this disorder [1], were included. Also included were subjects with insomnia and without any relevant sleep disorder as a control group without abnormal behaviors or movements during sleep. The developed method is robust for differentiation of people with iRBD not only from a control group but also from patients with movements during sleep which might be misdiagnosed as RBD based on clinical history or on video inspection only (in the absence of other PSG information). For the classification of both iRBD versus SRBD and iRBD versus INS/NRSD, the best average performances were achieved by combining short movements in the lower body with movements from other ROIs (head, hands and upper body for the classification iRBD vs. SRBD but only head and hands for the classification iRBD vs. INS/NRSD). It might seem counterintuitive that for the classification iRBD versus RLS/PLMS the best differentiation was achieved considering only movements in the lower body. A hypothesis is that leg movements during REM sleep might distinguish people with RBD even from those with RLS/PLMS, as in the latter group persistence of the physiological muscle atonia during REM sleep limits the appearance of leg movements during this sleep stage. Moreover, the algorithm identifies also very short movements of the lower extremities not fulfilling the criteria for candidate leg movements for PLMs, that is, those with a duration below 0.5 s. Due to the low number of patients with NREM parasomnia, performances were not provided for the differentiation of iRBD from this group alone.

The proposed analysis does not focus specifically on identification of RBD behaviors, which are needed to diagnose RBD [1, 5]. Therefore, the proposed technology cannot replace visual analysis of videos yet but could potentially be used as a supportive screening tool for clinicians in routine work. In future, the 3D video analysis could also be combined with automatic RWA quantification methods to provide a possibly even more accurate automatic screening of patients with RBD. In particular, in a recent study it was shown that automatic quantification of phasic activity in the flexor digitorum superficial muscles is a sensitive and specific method to identify patients with RBD [14], due to the low number of artifacts affecting the electromyographic recordings [29].

Future development of 3D video analysis in the context of RBD should aim at expanding the use of this technology in home settings without focusing on REM sleep only and without the need for manually selecting upper and lower body ROIs. Previous studies showing that people with iRBD have increased muscular activity also in NREM sleep [9, 30, 31] would support the hypothesis that identification of REM sleep might not be necessary, but further studies are needed to confirm this hypothesis. Furthermore, future studies should evaluate whether upper and lower ROIs can be automatically selected with segmentation [32] and pose estimation algorithms [33]. A future stand‐alone use of this technology at home as a screening tool for RBD would potentially allow identification of a larger number of people with iRBD, thus enlarging iRBD cohorts for future clinical trials.

As already outlined, one limitation of this work is that the thresholds to identify movements in the head, hands and upper body have not been optimized against manually annotated movements. Furthermore, another intrinsic limitation of video analysis is that movements occurring under the blankets which do not cause any blanket movement cannot be identified. For this reason, a combination of automatic electromyography and video analysis could possibly be the optimal solution to automatically identify RBD. Finally, the data were recorded all with the same TOF sensor and in the same sleep laboratory room. Future studies should investigate whether these aspects can have an influence on the results.

In conclusion, this study validated automatic 3D video analysis of short movements in head, hands, upper body and lower body regions as a new technology to identify people with iRBD. Compared to our previous study on 3D video analysis of the lower limbs [12], it is shown that including movements of the upper body, head and hands in the analysis significantly improved the classification performances. Furthermore, the proposed method is robust also against sleep disorders representing differential diagnoses of RBD. This technology could be used as a clinical supportive screening tool to identify people with iRBD and could potentially be applied as a screening instrument in the general population.

## CONFLICT OF INTERESTS STATEMENT

None.

## ETHICS STATEMENT

The ethical committee of the Medical University of Innsbruck approved this study.

## Supporting information


Appendix S1

## Data Availability

The data that support the findings of this study are available on request from the corresponding author. The data are not publicly available due to privacy or ethical restrictions.

## References

[1] American Academy of Sleep Medicine , ed. The International Classification of Sleep Disorders (ICSD‐3). 3rd ed. American Academy of Sleep Medicine; 2014.

[2] Högl B , Stefani A , Videnovic A . Idiopathic REM sleep behaviour disorder and neurodegeneration—an update. Nat Rev Neurol. 2018;14(1):40‐55. doi:10.1038/nrneurol.2017.157 29170501

[3] Postuma RB , Iranzo A , Hu M , et al. Risk and predictors of dementia and parkinsonism in idiopathic REM sleep behaviour disorder: a multicentre study. Brain. 2019;142(3):744‐759. doi:10.1093/brain/awz030 30789229 PMC6391615

[4] Videnovic A , Ju Y‐ES , Arnulf I , et al. Clinical trials in REM sleep behavioural disorder: challenges and opportunities. J Neurol Neurosurg Psychiatry. 2020;91(7):740‐749. doi:10.1136/jnnp-2020-322875 32404379 PMC7735522

[5] Cesari M , Heidbreder A , St Louis EK , et al. Video‐polysomnography procedures for diagnosis of rapid eye movement sleep behavior disorder (RBD) and the identification of its prodromal stages: guidelines from the international RBD study group. Sleep. 2022;43(3). doi:10.1093/sleep/zsab257 34694408

[6] Ferri R , Rundo F , Manconi M , et al. Improved computation of the atonia index in normal controls and patients with REM sleep behavior disorder. Sleep Med. 2010;11:947‐949. doi:10.1016/j.sleep.2010.06.003 20817596

[7] Mayer G , Kesper K , Ploch T , et al. Quantification of tonic and phasic muscle activity in REM sleep behavior disorder. J Clin Neurophysiol. 2008;25(1):48‐55. doi:10.1097/WNP.0b013e318162acd7 18303560

[8] Frauscher B , Gabelia D , Biermayr M , et al. Validation of an integrated software for the detection of rapid eye movement sleep behavior disorder. Sleep. 2014;37(10):1663‐1671. doi:10.5665/sleep.4076 25197814 PMC4173922

[9] Cesari M , Christensen JAE , Sixel‐Döring F , et al. Validation of a new data‐driven automated algorithm for muscular activity detection in REM sleep behavior disorder. J Neurosci Methods. 2019;312:53‐64. doi:10.1016/j.jneumeth.2018.11.016 30468824

[10] Frandsen R , Nikolic M , Zoetmulder M , Kempfner L , Jennum P . Analysis of automated quantification of motor activity in REM sleep behaviour disorder. J Sleep Res. 2015;24(5):583‐590. doi:10.1111/jsr.12304 25923472

[11] Kempfner J , Sorensen HBD , Nikolic M , Jennum P . Early automatic detection of Parkinson's disease based on sleep recordings. J Clin Neurophysiol. 2014;31:409‐415. 25271677 10.1097/WNP.0000000000000065

[12] Waser M , Stefani A , Holzknecht E , et al. Automated 3D video analysis of lower limb movements during REM sleep: a new diagnostic tool for isolated REM sleep behavior disorder. Sleep. 2020;43(11):zsaa100. doi:10.1093/sleep/zsaa100 32573731 PMC7658637

[13] Cesari M , Kohn B , Holzknecht E , et al. Automatic 3D video analysis of upper and lower body movements to identify isolated REM sleep behavior disorder: a pilot study. Annu Int Conf IEEE Eng Med and Biol Soc. 2021;2021:7050‐7053. 34892726 10.1109/EMBC46164.2021.9630011

[14] Cesari M , Heidbreder A , Gaig C , et al. Automatic analysis of muscular activity in the flexor digitorum superficialis muscles: a fast screening method for rapid eye movement sleep without atonia. Sleep. 2022;46. doi:10.1093/SLEEP/ZSAB299 PMC9995778 34984464

[15] Iranzo A , Frauscher B , Santos H , et al. Usefulness of the SINBAR electromyographic montage to detect the motor and vocal manifestations occurring in REM sleep behavior disorder. Sleep Med. 2011;12(3):284‐288. doi:10.1016/j.sleep.2010.04.021 21317034

[16] Frauscher B , Iranzo A , Gaig C , et al. Normative EMG values during REM sleep for the diagnosis of REM sleep behavior disorder. Sleep. 2012;35(6):835‐847. doi:10.5665/sleep.1886 22654203 PMC3353058

[17] Fernández‐Arcos A , Iranzo A , Serradell M , et al. Diagnostic value of isolated mentalis versus mentalis plus upper limb electromyography in idiopathic REM sleep behavior disorder patients eventually developing a neurodegenerative syndrome. Sleep. 2017;40(4):zsx025. doi:10.1093/sleep/zsx025 28201721

[18] Frauscher B , Brandauer E , Gschliesser V , et al. A descriptive analysis of neck myoclonus during routine polysomnography. Sleep. 2010;33(8):1091‐1096. 20815192 10.1093/sleep/33.8.1091 PMC2910539

[19] Berry RB , Brooks R , Gamaldo CE , et al. The AASM Manual for the Scoring of Sleep and Associated Events: Rules, Terminology and Technical Specifications: Version 2.6. American Academy of Sleep Medicine; 2020.

[20] Stefani A , Heidbreder A , Hackner H , Högl B . Validation of a leg movements count and periodic leg movements analysis in a custom polysomnography system. BMC Neurol. 2017;17(1):42. doi:10.1186/s12883-017-0821-6 28231845 PMC5324307

[21] Gall M , Garn H , Kohn B , et al. Automated detection of movements during sleep using a 3D time‐of‐flight camera: design and experimental evaluation. IEEE Access. Published online. 2020. doi:10.1109/ACCESS.2020.3001343

[22] Kohn B , Ruzicka L , Högl B , et al. TeaSpam: a novel method of TEmporal And SPAtial Movement encoding during sleep. Annu Int Conf IEEE Eng Med Bio Soc. 2022;2022:4222‐4225. 36085969 10.1109/EMBC48229.2022.9871521

[23] Frauscher B , Gschliesser V , Brandauer E , et al. Video analysis of motor events in REM sleep behavior disorder. Mov Disord. 2007;22(10):1464‐1470. doi:10.1002/mds.21561 17516467

[24] Nepozitek J , Unalp C , Dostalova S , et al. Systematic video‐analysis of motor events during REM sleep in idiopathic REM sleep behavior disorder, follow‐up and DAT‐SPECT. Sleep Med. 2021;28:132‐144. doi:10.1016/j.sleep.2021.04.033 33993030

[25] Garn H , Kohn B , Dittrich K , et al. 3D detection of periodic limb movements in sleep. Annu Int Conf Proc IEEE Eng Med Biol Soc. 2016;427‐430. doi:10.1109/EMBC.2016.7590731 28268364

[26] Garn H , Kohn B , Schmid F , et al. Contactless 3D detection of respiratory effort. IFMBE Proceedings. Vol 65. Springer Verlag; 2017:418–421. doi:10.1007/978-981-10-5122-7_105

[27] Iranzo A , Santamaría J . Severe obstructive sleep apnea/hypopnea mimicking REM sleep behavior disorder. Sleep. 2005;28(2):203‐206. doi:10.1093/sleep/28.2.203 16171244

[28] Gaig C , Iranzo A , Pujol M , Perez H , Santamaria J . Periodic limb movements during sleep mimicking REM sleep behavior disorder: a new form of periodic limb movement disorder. Sleep. 2017;40(3):zsw063. doi:10.1093/sleep/zsw063 28364416

[29] Cesari M , Heidbreder A , Bergmann M , Holzknecht E , Högl B , Stefani A . Flexor digitorum superficialis muscular activity is more reliable than mentalis muscular activity for rapid eye movement sleep without atonia quantification: a study of interrater reliability for artifact correction in the context of semiautomated scoring of rapid eye movement sleep without atonia. Sleep. 2021;44(9):zsab094. doi:10.1093/sleep/zsab094 33842971

[30] Schenck CH , Hurwitz TD , Mahowald MW . REM sleep behaviour disorder: an update on a series of 96 patients and a review of the world literature. J Sleep Res. 1993;2(4):224‐231. doi:10.1111/j.1365-2869.1993.tb00093.x 10607098

[31] Cesari M , Christensen JAE , Sorensen HBD , et al. External validation of a data‐driven algorithm for muscular activity identification during sleep. J Sleep Res. 2019;28(6):e12868. doi:10.1111/jsr.12868 31131530

[32] Goldstein Y , Schätz M , Avigal M . Chest area segmentation in 3D images of sleeping patients. Med Biol Eng Comput. 2022;60(8):2159‐2172. doi:10.1007/S11517-022-02577-1/TABLES/3 35644821

[33] Liu S , Yin Y , Ostadabbas S . In‐bed pose estimation: deep learning with shallow dataset. IEEE J Transl Eng Heal Med. 2019;7:7. doi:10.1109/JTEHM.2019.2892970 PMC6360998 30792942

