# Supplementary material for: Improved automatic identification of isolated rapid eye movement sleep behavior disorder with a 3D time‐of‐flight camera
Source: Eur J Neurol. 2023 May 23;30(8):2206–14. doi: 10.1111/ene.15822 (PMC10947372; doi:10.1111/ene.15822)
Supplement: Supplementary file 1 — Appendix S1 [file ENE-30-2206-s001.pdf]

SUPPORTING INFORMATION

Improved automatic identification of isolated rapid eye movement sleep behavior disorder with a 3D time-of-flight camera

Matteo Cesari[1], Laurenz Ruzicka[2], Birgit Högl[1], Abubaker Ibrahim[1], Evi Holzknecht[1], Anna Heidbreder[1], Melanie Bergmann[1], Elisabeth Brandauer[1], Heinrich Garn[2], Bernhard Kohn[2], Ambra Stefani[1]

[1]Department of Neurology, Medical University of Innsbruck, Innsbruck, Austria

[2]Competence Unit Sensing and Vision Solutions, AIT Austrian Institute of Technology GmbH, Vienna, Austria.

Corresponding author

Ambra Stefani, MD, PhD

Department of Neurology

Medical University of Innsbruck

Anichstrasse 35, 6020 Innsbruck, Austria

e-mail: ambra.stefani@i-med.ac.at

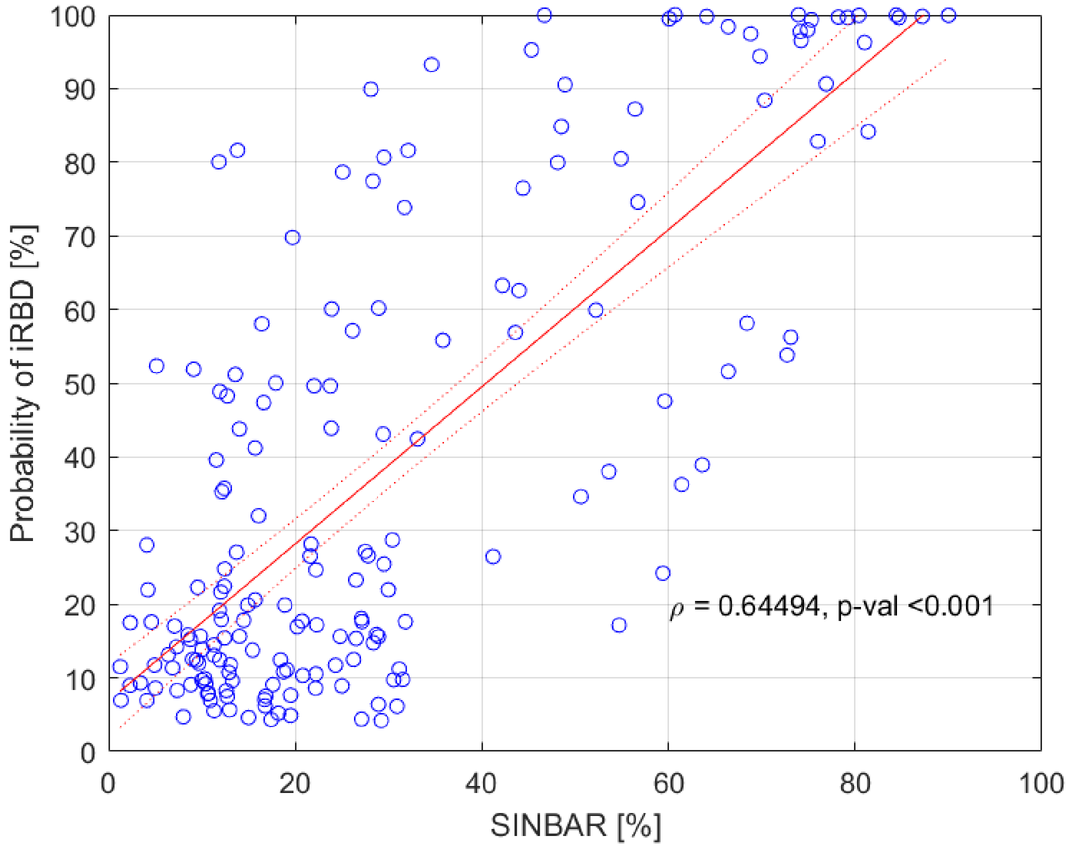

Figure S1: Correlation between the SINBAR RWA index and test probability of iRBD from logistic regression models. The test probability of the 10 runs of classification considering short movements (0.1s-2s) in the four ROIs were averaged to obtain the values reported in the y-axis. The tick red line shows the fit and the dashed lines the 95% confidence interval of the fit.

Legend: iRBD: isolated REM sleep behavior disorder; p-val: p value, REM: rapid eye movement; ROI: region of interest; RWA: REM sleep without atonia; SINBAR: sleep Innsbruck Barcelona; ρ = Spearman correlation coefficient.

Table S1: Demographic and sleep information of iRBD and sleep-related breathing disorder patients. Values are shown as mean±standard deviation if normally distributed and as median [interquartile range] otherwise. For normally distributed variables, t-tests were used to compare groups, otherwise Mann-U-Whitney tests were employed. Categorical variables were compared with chi-square tests. Significant p-values (<0.05) are highlighted in bold.

**Parameter**	**iRBD**	**Sleep related breathing disorder**	**p-value**
Number	53	51	-
Males (%)	84.9	74.5	0.187
Age (years)	65.5±9.5	58.6±11.8	**0.001**
REM duration (min)	66.1±31.4	71.5±36.1	0.418
AHI (/h)	6.8 [2.7-13.2]	22.6 [11.9-54.3]	**<0.001**
AHI in REM (/h)	7.6 [1.7-14.0]	27.6 [8.3-48.0]	**<0.001**
PLMS index (/h)	28.1 [16.5-58.4]	21.5 [10.1-33.7]	**0.027**
PLMS index in REM (/h)	38.0 [19.0-73.0]	2.0 [0.0-9.8]	**<0.001**
PAP therapy during v-PSG (%)	34.0	23.5	0.240
Taking antidepressants (%)	47.2	15.7	**<0.001**
SINBAR (%)	61.4±16.0	18.8±7.8	**<0.001**

Legend: AHI: apnea hypopnea index; iRBD: isolated REM sleep behavior disorder; PAP: positive air pressure; PLMS: periodic limb movement during sleep; REM: rapid eye movement; SINBAR: sleep Innsbruck Barcelona.

Table S2: Demographic and sleep information of iRBD and patients with restless legs syndrome. Values are shown as mean±standard deviation if normally distributed and as median [interquartile range] otherwise. For normally distributed variables, t-tests were used to compare groups, otherwise Mann-U-Whitney tests were employed. Categorical variables were compared with chi-square tests. Significant p-values (<0.05) are highlighted in bold.

Parameter	iRBD	Restless legs syndrome	p-value
Number	53	20	-
Males (%)	84.9	50.0	**0.002**
Age (years)	65.5±9.5	60.3±8.6	**0.036**
REM duration (min)	66.1±31.4	59.6±30.5	0.427
AHI (/h)	6.8 [2.7-13.2]	8.5 [3.7-13.1]	0.549
AHI in REM (/h)	7.6 [1.7-14.0]	10.9 [2.8-20.4]	0.389
PLMS index (/h)	28.1 [16.5-58.4]	30.1 [9.4-74.3]	0.720
PLMS index in REM (/h)	38.0 [19.0-73.0]	4.0 [1.0-14.5]	**<0.001**
PAP therapy during v-PSG (%)	34.0	5.0	**0.012**
Taking antidepressants (%)	47.2	20.0	**0.034**
SINBAR (%)	61.4 [48.4-74.4]	11.5 [8.9, 14.2]	**<0.001**

Legend: AHI: apnea hypopnea index; iRBD: isolated REM sleep behavior disorder; PAP: positive air pressure; PLMS: periodic limb movement during sleep; REM: rapid eye movement; SINBAR: sleep Innsbruck Barcelona.

Table S3: Demographic and sleep information of iRBD and patients with PLMS without any associated sleep disorder. Values are shown as mean±standard deviation if normally distributed and as median [interquartile range] otherwise. For normally distributed variables, t-tests were used to compare groups, otherwise Mann-U-Whitney tests were employed. Categorical variables were compared with chi-square tests. Significant p-values (<0.05) are highlighted in bold.

**Parameter**	**iRBD**	**PLMS without any associated sleep disorder**	**p-value**
Number	53	12	-
Males (%)	84.9	75	0.409
Age (years)	65.5±9.5	51.9±11.4	**<0.001**
REM duration (min)	66.1±31.4	73.0±25.6	0.482
AHI (/h)	6.8 [2.7-13.2]	2.9 [1.0, 7.2]	**0.024**
AHI in REM (/h)	7.6 [1.7-14.0]	1.0 [0.3-3.8]	**0.003**
PLMS index (/h)	28.1 [16.5-58.4]	36.4 [18.15-53.3]	0.879
PLMS index in REM (/h)	38.0 [19.0-73.0]	8.0 [1.0-21.5]	**0.001**
PAP therapy during v-PSG (%)	34.0	0.0	**0.018**
Taking antidepressants (%)	47.2	8.3	**0.013**
SINBAR (%)	61.4±16.0	18.0±9.1	**<0.001**

Legend: AHI: apnea hypopnea index; iRBD: isolated REM sleep behavior disorder; PAP: positive air pressure; PLMS: periodic limb movement during sleep; REM: rapid eye movement; SINBAR: sleep Innsbruck Barcelona.

Table S4: Demographic and sleep information of iRBD and patients with insomnia. Values are shown as mean±standard deviation if normally distributed and as median [interquartile range] otherwise. For normally distributed variables, t-tests were used to compare groups, otherwise Mann-U-Whitney tests were employed. Categorical variables were compared with chi-square tests. Significant p-values (<0.05) are highlighted in bold.

**Parameter**	**iRBD**	**Insomnia**	**p-value**
Number	53	20	-
Males (%)	84.9	45.0	**<0.001**
Age (years)	65.5±9.5	54.5 [49.5, 57.0]	**<0.001**
REM duration (min)	66.1±31.4	64.2±21.5	0.804
AHI (/h)	6.8 [2.7-13.2]	1.0 [0.1-2.8]	**<0.001**
AHI in REM (/h)	7.6 [1.7-14.0]	1.1 [0.0-4.0]	**<0.001**
PLMS index (/h)	28.1 [16.5-58.4]	2.8 [0.9-5.7]	**<0.001**
PLMS index in REM (/h)	38.0 [19.0-73.0]	0.5 [0.0-5.5]	**<0.001**
PAP therapy during v-PSG (%)	34.0	0.0	**0.003**
Taking antidepressants (%)	47.2	25.0	0.086
SINBAR (%)	61.4±16.0	14.0±8.4	**<0.001**

Legend: AHI: apnea hypopnea index; iRBD: isolated REM sleep behavior disorder; PAP: positive air pressure; PLMS: periodic limb movement during sleep; REM: rapid eye movement; SINBAR: sleep Innsbruck Barcelona.

Table S5: Demographic and sleep information of iRBD and patients with non-REM parasomnia. Values are shown as mean±standard deviation if normally distributed and as median [interquartile range] otherwise. For normally distributed variables, t-tests were used to compare groups, otherwise Mann-U-Whitney tests were employed. Categorical variables were compared with chi-square tests. Significant p-values (<0.05) are highlighted in bold.

**Parameter**	**iRBD**	**Non-REM parasomnia**	**p-value**
Number	53	6	-
Males (%)	84.9	66.7	0.259
Age (years)	65.5±9.5	33.3±8.1	**<0.001**
REM duration (min)	66.1±31.4	87.9±31.9	0.113
AHI (/h)	6.8 [2.7-13.2]	0.7 [0.4-2.0]	**0.033**
AHI in REM (/h)	7.6 [1.7-14.0]	2.0 [0.9-3.0]	0.175
PLMS index (/h)	28.1 [16.5-58.4]	11.3 [3.2, 25.7]	**0.042**
PLMS index in REM (/h)	38.0 [19.0-73.0]	0.0 [0.0-1.0]	**<0.001**
PAP therapy during v-PSG (%)	34.0	0.0	0.086
Taking antidepressants (%)	47.2	16.7	0.154
SINBAR (%)	61.4±16.0	14.5±8.5	**<0.001**

Legend: AHI: apnea hypopnea index; iRBD: isolated REM sleep behavior disorder; PAP: positive air pressure; PLMS: periodic limb movement during sleep; REM: rapid eye movement; SINBAR: sleep Innsbruck Barcelona.

Table S6: Demographic and sleep information of iRBD and subjects without any relevant sleep disorder. Values are shown as mean±standard deviation if normally distributed and as median [interquartile range] otherwise. For normally distributed variables, t-tests were used to compare groups, otherwise Mann-U-Whitney tests were employed. Categorical variables were compared with chi-square tests. Significant p-values (<0.05) are highlighted in bold.

Parameter	iRBD	No relevant sleep disorder	p-value
Number	53	19	-
Males (%)	84.9	47.4	**0.001**
Age (years)	65.5±9.5	45.2±15.9	**<0.001**
REM duration (min)	66.1±31.4	84.0±29.5	**0.034**
AHI (/h)	6.8 [2.7-13.2]	3.6 [1.7-5.2]	**0.035**
AHI in REM (/h)	7.6 [1.7-14.0]	4.9 [0.8-8.0]	0.177
PLMS index (/h)	28.1 [16.5-58.4	2.3 [1.0-8.8]	**<0.001**
PLMS index in REM (/h)	38.0 [19.0-73.0]	0.0 [0.0-3.0]	**<0.001**
PAP therapy during v-PSG (%)	34.0	0.0	**0.003**
Taking antidepressants (%)	47.2	15.8	**0.017**
SINBAR (%)	61.4±16.0	18.4±8.0	**<0.001**

Legend: AHI: apnea hypopnea index; iRBD: isolated REM sleep behavior disorder; PAP: positive air pressure; PLMS: periodic limb movement during sleep; REM: rapid eye movement; SINBAR: sleep Innsbruck Barcelona.

Table S7: Performances in the training folds for the classification iRBD vs no-RBD. The performance measures are reported for each interval duration and when considering as predictor features the 3D rate and 3D ratio from: i) head region of interest (ROI) only (HE), ii) hands ROI only (HAs), iii) upper body ROI only (UB), iv) lower body ROI only (LB), v) head, hands and upper body ROIs (HE+HAs+UB), vi) head, hands and lower body ROIs (HE+HAs+LB), and vii) head, hands, upper body and lower body ROIs (HE+HAs+UB+LB). The performances are reported as mean (μ) and standard deviation (σ) across the 10 runs of 10-fold-cross-validation.

Predictor features from ROI(s)	Measure	Interval					
		0.1s-2s		2s-15s		15s-300s	
		μ	σ	μ	σ	μ	σ
HE	Accuracy	0.761	0.01	0.62	0.017	0.691	0.009
	F1-score	0.607	0.016	0.411	0.026	0.282	0.023
	Sensitivity	0.63	0.024	0.455	0.035	0.208	0.019
	Specificity	0.816	0.012	0.688	0.021	0.891	0.009
	PPV	0.586	0.016	0.376	0.022	0.44	0.033
	NPV	0.842	0.008	0.753	0.012	0.731	0.005
HAs	Accuracy	0.698	0.014	0.631	0.01	0.707	0.002
	F1-score	0.506	0.02	0.378	0.017	0	0
	Sensitivity	0.528	0.022	0.383	0.019	0	0
	Specificity	0.768	0.017	0.733	0.011	1	0
	PPV	0.486	0.022	0.373	0.016	0	0
	NPV	0.797	0.009	0.742	0.007	0.707	0.002
UB	Accuracy	0.819	0.011	0.675	0.012	0.622	0.017
	F1-score	0.667	0.021	0.491	0.017	0.379	0.019
	Sensitivity	0.618	0.021	0.536	0.023	0.393	0.024
	Specificity	0.902	0.01	0.732	0.015	0.717	0.024

	PPV	0.724	0.025	0.453	0.017	0.366	0.022
	NPV	0.851	0.008	0.792	0.009	0.74	0.009
**LB**	Accuracy	0.848	0.013	0.844	0.009	0.664	0.013
	F1-score	0.763	0.019	0.729	0.016	0.476	0.019
	Sensitivity	0.835	0.019	0.717	0.016	0.521	0.021
	Specificity	0.853	0.013	0.897	0.009	0.722	0.014
	PPV	0.702	0.021	0.743	0.02	0.438	0.02
	NPV	0.926	0.009	0.884	0.006	0.785	0.009
**HE+HAs+UB**	Accuracy	0.823	0.01	0.677	0.019	0.656	0.018
	F1-score	0.685	0.019	0.487	0.028	0.358	0.055
	Sensitivity	0.658	0.024	0.523	0.031	0.332	0.072
	Specificity	0.891	0.01	0.741	0.018	0.791	0.037
	PPV	0.715	0.021	0.456	0.028	0.395	0.039
	NPV	0.863	0.008	0.79	0.013	0.742	0.015
**HE+HAs+LB**	Accuracy	0.863	0.012	0.85	0.012	0.657	0.014
	F1-score	0.785	0.018	0.741	0.022	0.463	0.022
	Sensitivity	0.849	0.018	0.733	0.025	0.506	0.026
	Specificity	0.869	0.012	0.898	0.01	0.719	0.014
	PPV	0.729	0.021	0.749	0.023	0.427	0.021
	NPV	0.933	0.008	0.89	0.01	0.779	0.011
**HE+HAs+UB+LB**	Accuracy	0.878	0.011	0.852	0.012	0.664	0.015
	F1-score	0.802	0.017	0.747	0.019	0.469	0.022
	Sensitivity	0.845	0.016	0.744	0.021	0.507	0.026
	Specificity	0.892	0.013	0.897	0.012	0.729	0.014
	PPV	0.764	0.023	0.75	0.024	0.436	0.022
	NPV	0.933	0.007	0.894	0.008	0.781	0.01

Legend: NPV: negative predictive value; PPV: positive predictive value.

Table S8: Test performances for the classification iRBD vs no-RBD. The performance measures are reported for each interval duration and when considering as predictor features the 3D rate and 3D ratio from: i) head region of interest (ROI) only (HE), ii) hands ROI only (HAs), iii) upper body ROI only (UB), iv) lower body ROI only (LB), v) head, hands and upper body ROIs (HE+HAs+UB), vi) head, hands and lower body ROIs (HE+HAs+LB), and vii) head, hands, upper body and lower body ROIs (HE+HAs+UB+LB).

Predictor features from ROI(s)	Measure	Interval					
		0.1s-2s		2s-15s		15s-300s	
		μ	σ	μ	σ	μ	σ
**HE**	Accuracy	0.762	0.007	0.614	0.01	0.691	0
	F1-score	0.609	0.01	0.398	0.018	0.282	0
	Sensitivity	0.632	0.013	0.436	0.023	0.208	0
	Specificity	0.816	0.008	0.688	0.009	0.891	0
	PPV	0.587	0.012	0.367	0.015	0.44	0
	NPV	0.843	0.005	0.747	0.009	0.731	0
**HAs**	Accuracy	0.696	0.006	0.634	0.006	0.707	0
	F1-score	0.499	0.009	0.382	0.014	0	0
	Sensitivity	0.517	0.013	0.387	0.018	0	0
	Specificity	0.77	0.008	0.737	0.006	1	0
	PPV	0.482	0.009	0.378	0.011	0	0
	NPV	0.794	0.004	0.744	0.006	0.707	0
**UB**	Accuracy	0.819	0.003	0.67	0.007	0.617	0.01
	F1-score	0.667	0.006	0.484	0.012	0.374	0.011
	Sensitivity	0.619	0.008	0.528	0.015	0.391	0.009
	Specificity	0.902	0.004	0.728	0.005	0.71	0.012
	PPV	0.723	0.008	0.446	0.01	0.358	0.013
	NPV	0.851	0.003	0.789	0.006	0.738	0.005

**LB**	Accuracy	0.847	0.004	0.844	0.006	0.662	0.004
	F1-score	0.762	0.007	0.729	0.007	0.474	0.007
	Sensitivity	0.836	0.013	0.717	0	0.521	0.01
	Specificity	0.852	0	0.897	0.008	0.72	0.005
	PPV	0.7	0.003	0.742	0.015	0.435	0.006
	NPV	0.926	0.005	0.884	0.001	0.784	0.004
**HE+HAs+UB**	Accuracy	0.816	0.007	0.665	0.011	0.634	0.009
	F1-score	0.673	0.011	0.469	0.018	0.31	0.02
	Sensitivity	0.647	0.009	0.506	0.025	0.281	0.021
	Specificity	0.886	0.008	0.73	0.012	0.78	0.009
	PPV	0.702	0.017	0.437	0.016	0.346	0.019
	NPV	0.858	0.004	0.781	0.009	0.724	0.006
**HE+HAs+LB**	Accuracy	0.849	0.008	0.831	0.008	0.648	0.008
	F1-score	0.764	0.011	0.708	0.015	0.451	0.009
	Sensitivity	0.832	0.011	0.698	0.022	0.494	0.012
	Specificity	0.856	0.009	0.887	0.008	0.712	0.011
	PPV	0.706	0.014	0.719	0.014	0.415	0.01
	NPV	0.925	0.005	0.877	0.008	0.773	0.005
**HE+HAs+UB+LB**	Accuracy	0.866	0.007	0.835	0.008	0.649	0.007
	F1-score	0.783	0.01	0.714	0.015	0.446	0.017
	Sensitivity	0.828	0.011	0.702	0.021	0.483	0.025
	Specificity	0.881	0.01	0.891	0.006	0.717	0.01
	PPV	0.743	0.015	0.727	0.013	0.414	0.012
	NPV	0.925	0.004	0.878	0.008	0.77	0.008

Legend: NPV: negative predictive value; PPV: positive predictive value.

Table S9: P-values from the comparison of performances considering different movement lengths for the classification iRBD vs no-RBD. The table reports the corrected p-values obtained from comparing test accuracy and F1-scores of classifiers trained and tested using movements of different durations: short movements (0.1s-2s), medium duration movements (2s-15s) and long movements (15s-300s). The p-values are reported for classifiers trained and tested when considering as predictor features the 3D rate and 3D ratio from: i) head region of interest (ROI) only (HE), ii) hands ROI only (HAs), iii) upper body ROI only (UB), iv) lower body ROI only (LB), v) head, hands and upper body ROIs (HE+HAs+UB), vi) head, hands and lower body ROIs (HE+HAs+LB), and vii) head, hands, upper body and lower body ROIs (HE+HAs+UB+LB). P-values<0.05 are considered significant.

**ROI**	**0.1s-2s compared to 2s-15s**		**01.s-2s compared to 15s-300s**		**2s-15s compared to 15s-300s**	
	P-value for accuracy	P-value for F1-score	P-value for accuracy	P-value for F1-score	P-value for accuracy	P-value for F1-score
**HE**	0.004	0.004	0.004	0.004	0.004	0.004
**HAs**	0.005	0.004	0.007	0.004	0.005	0.004
**UB**	0.004	0.004	0.004	0.004	0.004	0.004
**LB**	0.516	0.004	0.005	0.004	0.005	0.004
**HE+ HAs + UB**	0.004	0.004	0.004	0.004	0.004	0.004
**HE+ HAs + LB**	0.007	0.004	0.005	0.004	0.005	0.004
**HE+ HAs + UB + LB**	0.004	0.004	0.004	0.004	0.004	0.004

Table S10: P-values from the comparison of performances considering different ROIs and short movements (classification iRBD vs no-RBD). The table reports the corrected p-values obtained from comparing test accuracy and F1-scores of classifiers trained and tested using short movements when considering as predictor features the 3D rate and 3D ratio from: i) head region of interest (ROI) only (HE), ii) hands ROI only (HAs), iii) upper body ROI only (UB), iv) lower body ROI only (LB), v) head, hands and upper body ROIs (HE+HAs+UB), vi) head, hands and lower body ROIs (HE+HAs+LB), and vii) head, hands, upper body and lower body ROIs (HE+HAs+UB+LB). P-values<0.05 are considered significant. n.s. = non significant.

Comparison	P-value for accuracy	P-value for F1-score
**HE compared to HAs**	0.008	0.009
**HE compared to UB**	0.008	0.009
**HE compared to LB**	0.008	0.009
**HE compared to HE + HAs + UB**	0.008	0.009
**HE compared to HE + HAs + LB**	0.008	0.009
**HE compared to HE + HAs + UB +LB**	0.008	0.009
**HAs compared to UB**	0.008	0.009
**HAs compared to LB**	0.008	0.009
**HAs compared to HE + HAs + UB**	0.008	0.009
**HAs compared to HE + HAs + LB**	0.008	0.009
**HAs compared to HE + HAs + UB +LB**	0.008	0.009
**UB compared to LB**	0.008	0.009
**UB compared to HE + HAs + UB**	n.s.	n.s.
**UB compared to HE + HAs + LB**	0.008	0.009
**UB compared to HE + HAs + UB +LB**	0.008	0.009
**LB compared to HE + HAs + UB**	0.008	0.009
**LB compared to HE + HAs + LB**	n.s.	n.s.
**LB compared to HE + HAs + UB +LB**	0.008	0.016

**HE + HAs + UB compared to HE + HAs + LB**	0.008	0.009
**HE + HAs + UB compared to HE + HAs + UB +LB**	0.008	0.009
**HE + HAs + LB compared to HE + HAs + UB +LB**	0.016	0.016

Table S11: Test performances for the classifications: iRBD vs SRBD, iRBD vs RLS/PLMS and iRBD vs INS/NRSD. The performance measures are reported only for the interval 0.1s-2s and when considering as predictor features the 3D rate and 3D ratio from: i) head region of interest (ROI) only (HE), ii) hands ROI only (HAs), iii) upper body ROI only (UB), iv) lower body ROI only (LB), v) head, hands and upper body ROIs (HE+HAs+UB), vi) head, hands and lower body ROIs (HE+HAs+LB), and vii) head, hands, upper body and lower body ROIs (HE+HAs+UB+LB).

Predictor features from ROI(s)	Measure	iRBD vs...					
		SRBD		RLS/PLMS		INS/NRSD	
		μ	σ	μ	σ	μ	σ
**HE**	Accuracy	0.761	0.008	0.664	0.011	0.7	0.008
	F1-score	0.729	0.011	0.701	0.011	0.708	0.009
	Sensitivity	0.632	0.013	0.632	0.013	0.632	0.013
	Specificity	0.894	0.01	0.716	0.023	0.792	0.008
	PPV	0.861	0.012	0.787	0.014	0.805	0.006
	NPV	0.701	0.008	0.54	0.011	0.613	0.008
**HAs**	Accuracy	0.623	0.008	0.593	0.008	0.665	0.01
	F1-score	0.583	0.01	0.613	0.011	0.64	0.012
	Sensitivity	0.517	0.013	0.517	0.013	0.517	0.013
	Specificity	0.733	0.014	0.719	0	0.867	0.011
	PPV	0.668	0.011	0.753	0.005	0.841	0.012
	NPV	0.594	0.007	0.473	0.007	0.569	0.008
**UB**	Accuracy	0.743	0.008	0.702	0.006	0.77	0.005
	F1-score	0.711	0.009	0.722	0.005	0.756	0.006
	Sensitivity	0.619	0.008	0.619	0.008	0.619	0.008
	Specificity	0.873	0.01	0.841	0.018	0.974	0
	PPV	0.835	0.012	0.866	0.012	0.97	0
	NPV	0.688	0.006	0.571	0.005	0.653	0.005
**LB**	Accuracy	0.811	0.006	0.839	0.008	0.884	0.007

	F1-score	0.818	0.007	0.866	0.007	0.892	0.008
	Sensitivity	0.836	0.013	0.836	0.013	0.836	0.013
	Specificity	0.784	0	0.844	0	0.949	0
	PPV	0.801	0.002	0.899	0.001	0.957	0.001
	NPV	0.821	0.011	0.757	0.014	0.81	0.012
**HE+HAs+UB**	Accuracy	0.747	0.011	0.709	0.01	0.786	0.005
	F1-score	0.723	0.011	0.735	0.008	0.777	0.007
	Sensitivity	0.647	0.009	0.647	0.009	0.647	0.009
	Specificity	0.851	0.017	0.813	0.021	0.974	0
	PPV	0.819	0.017	0.851	0.014	0.972	0
	NPV	0.699	0.008	0.582	0.009	0.67	0.006
**HE+HAs+LB**	Accuracy	0.824	0.01	0.818	0.011	0.888	0.007
	F1-score	0.828	0.009	0.851	0.009	0.895	0.007
	Sensitivity	0.832	0.011	0.832	0.011	0.832	0.011
	Specificity	0.816	0.017	0.794	0.016	0.964	0.013
	PPV	0.824	0.013	0.87	0.01	0.969	0.011
	NPV	0.824	0.01	0.741	0.015	0.809	0.01
**HE+HAs+UB+LB**	Accuracy	0.839	0.006	0.834	0.01	0.884	0.009
	F1-score	0.84	0.006	0.862	0.008	0.891	0.008
	Sensitivity	0.828	0.011	0.828	0.011	0.828	0.011
	Specificity	0.851	0.014	0.844	0.021	0.959	0.013
	PPV	0.853	0.011	0.898	0.012	0.965	0.011
	NPV	0.827	0.008	0.748	0.013	0.804	0.01

Legend: NPV: negative predictive value; PPV: positive predictive value

Table S12: P-values from the comparison of performances considering different groups. The table reports the corrected p-values obtained from comparing test accuracy and F1-scores of classifiers trained and tested using short movements (0.1s-2s) and for different classification problems: i) iRBD vs SRBD, ii) iRBD vs RLS/PLMS and iii) iRBD vs INS/NRSD. The p-values are reported for classifiers trained and tested when considering as predictor features the 3D rate and 3D ratio from: i) head region of interest (ROI) only (HE), ii) hands ROI only (HAs), iii) upper body ROI only (UB), iv) lower body ROI only (LB), v) head, hands and upper body ROIs (HE+HAs+UB), vi) head, hands and lower body ROIs (HE+HAs+LB), and vii) head, hands, upper body and lower body ROIs (HE+HAs+UB+LB). P-values<0.05 are considered significant.

**ROI**	**iRBD vs SRBD compared to iRBD vs RLS/PLMS**		**iRBD vs SRBD compared to iRBD vs INS/NRSD**		**iRBD vs RLS/PLMS compared to iRBD vs INS/NRSD**	
	Accuracy	F1-score	Accuracy	F1-score	Accuracy	F1-score
**HE**	0.004	0.005	0.004	0.005	0.004	0.014
**HAs**	0.004	0.004	0.004	0.004	0.004	0.004
**UB**	0.004	0.014	0.004	0.005	0.004	0.005
**LB**	0.004	0.004	0.004	0.004	0.004	0.004
**HE+ HAs + UB**	0.004	0.014	0.004	0.005	0.004	0.005
**HE+ HAs + LB**	0.193	0.004	0.005	0.004	0.005	0.004
**HE+ HAs + UB + LB**	0.140	0.004	0.005	0.004	0.005	0.004

Legend: INS: insomnia; iRBD: isolated rapid eye movement sleep behavior disorder; NRSD: non-relevant sleep disorder; PLMS: periodic limb movements during sleep; RLS: restless legs syndrome; SRBD: sleep-related breathing disorder.

Table S13: Details of the linear regression model evaluating the influence of age, sex and groups on the classification. The test probability of the 10 runs of classification considering short movements (0.1s-2s) in the four ROIs were averaged to obtain the values of p(iRBD). The following linear regression model was fit: log(p(iRBD)) ~ 1+age+sex + iRBD + SRBD + RLS + PLMS + Insomnia + NREM parasomnia. The sex and groups were considered categorical variables. The log-transformation for p(iRBD) was done to ensure normal residuals.

Variable	Estimate	t-statistic	p-value
**Intercept**	-2.167	-8.142	**<0.001**
**Age**	0.002	0.472	0.638
**Sex (male)**	0.071	0.619	0.537
**iRBD**	1.626	7.789	**<0.001**
**SRBD**	0.278	1.423	0.157
**RLS**	0.282	1.235	0.218
**PLMS**	0.558	2.195	**0.029**
**Insomnia**	0.133	0.602	0.548
**NREM parasomnia**	-0.040	-0.124	0.902
